# Virulence and resistance profiling of *Staphylococcus aureus* isolated from subclinical bovine mastitis in the Pakistani Pothohar region

**DOI:** 10.1038/s41598-024-65448-9

**Published:** 2024-06-24

**Authors:** Muhammad Armaghan Shahzad, Arfan Yousaf, Aitezaz Ahsan, Hamid Irshad, Aayesha Riaz, Asghar Khan, Inayat Ullah, Sadia Sattar, Nazish Bostan, Sundus Javed

**Affiliations:** 1 Department of Biosciences, COMSATS University Islamabad, Park Road, Tarlai Kalan, Islamabad, Pakistan; 2 Department of Clinical Studies, Faculty of Veterinary and Animal Sciences, PMAS Arid Agriculture University, Rawalpindi, Pakistan; 3 Animal Health Laboratories, Animal Sciences Institute, NARC, Islamabad, Pakistan; 4 Department of Pathobiology, Faculty of Veterinary and Animal Sciences, PMAS Arid Agriculture University, Rawalpindi, Pakistan; 5 Department of Clinical Medicine and Surgery, Faculty of Veterinary Science, University of Agriculture Faisalabad, Faisalabad, Pakistan

**Keywords:** Subclinical mastitis, *Staphylococcus aureus*, MRSA, SCCmec typing, Virulence genes, Antibiotic resistance, Microbial genetics, Pathogens, Diseases, Pathogenesis

## Abstract

Mastitis is considered one of the most widespread infectious disease of cattle and buffaloes, affecting dairy herds. The current study aimed to characterize the *Staphylococcus aureus* isolates recovered from subclinical mastitis animals in Pothohar region of the country. A total of 278 milk samples from 17 different dairy farms around two districts of the Pothohar region, Islamabad and Rawalpindi, were collected and screened for sub clinical mastitis using California Mastitis Test. Positive milk samples were processed for isolation of *Staphylococcus aureus* using mannitol salt agar. The recovered isolates were analyzed for their antimicrobial susceptibility and virulence genes using disc diffusion and PCR respectively. 62.2% samples were positive for subclinical mastitis and in total 70 *Staphylococcus aureus* isolates were recovered. 21% of these isolates were determined to be methicillin resistant, carrying the *mecA* gene. *S. aureus* isolates recovered during the study were resistant to all first line therapeutic antibiotics and in total 52% isolates were multidrug resistant. SCCmec typing revealed MRSA SCCmec types IV and V, indicating potential community-acquired MRSA (CA-MRSA) transmission. Virulence profiling revealed high prevalence of key genes associated with adhesion, toxin production, and immune evasion, such as *hla, hlb, clfA, clfB* and *cap5*. Furthermore, the Panton-Valentine leukocidin (PVL) toxin, that is often associated with recurrent skin and soft tissue infections, was present in 5.7% of isolates. In conclusion, the increased prevalence of MRSA in bovine mastitis is highlighted by this study, which also reveals a variety of virulence factors in *S. aureus* and emphasizes the significance of appropriate antibiotic therapy in combating this economically burdensome disease.

## Introduction

Mastitis is considered one of most widespread bovine infections, affecting dairy herds but the prevalence varies globally. In this disease, parenchyma of mammary glands gets inflamed causing pathological changes in glandular tissues leading to physical, chemical, and usually bacteriological, changes in milk composition^1^. Studies have indicated that more than 137 microbes can act as etiological agents of mastitis^2^.

*Staphylococcus aureus* is one of the most common etiological agents associated with mastitis ^3^. Prevalence of *Staphylococcus aureus*-associated mastitis was found to be 32%, 27%, 23%, 51%, 13%, 31%, and 41% from Africa, Asia, Europe, Latin America, North America, Oceania, and India, respectively. In Pakistan, different studies report the overall mastitis prevalence rates, ranging from 42 to 70%^4–7^. While mastitis in cattle causes major productivity losses, subclinical mastitis is considered a major threat due to the absence of any visible symptoms. In subclinical mastitis there are no visible abnormalities of either the milk or the udder, which may mask the disease incidence^8^. In India, the prevalence rate of subclinical mastitis was reported as low as 9.8% to as high as 86%^9^. Meanwhile, in Pakistan, the prevalence rate of subclinical mastitis was estimated to be between 29 and 57%^10–12^.

Mastitis stands out as one of the most costly diseases in terms of losses incurred in dairy production. Globally, it contributes to an annual financial loss of approximately 33 billion USD^13^. In Pakistan, research by Fareed et al. indicates that mastitis alone accounts for 17% of the total economic losses resulting from animal diseases^14^. *S. aureus* is responsible for more than 80% of subclinical mastitis reported^15^. *S. aureus* produces several virulence factors that promote its invasive ability and aid in soft tissue penetration that helps in pathogenesis^16^. In addition to that the infection, once detected, can be difficult to treat due to increasing resistance against mainstay antimicrobial drugs. Of particular concern is the emergence of methicillin-resistant staphylococci, characterized by the presence of the *mecA* gene, posing a significant threat to both public and animal health. Methicillin resistant *Staphylococcus aureus* (MRSA) plays a vital role in the infection of the skin and soft tissue^17^.

While MRSA prevalence is widespread in humans, its occurrence in livestock appears to be relatively limited. *S. aureus* possesses various virulence factors associated with structural components as well as several secretory virulence factors including enterotoxins, tsst1, exfoliatin, and hemolysins as well as enzymes. The combination of these virulence properties dictates disease chronicity and severity. Some of the immune evasive mechanisms employed by the bacterium may underline the sub-clinical infection status maintained by *S. aureus*. Limited studies from Pakistan have focused on the virulence properties of this pathogen in livestock setups. Molecular analysis has been limited to the detection of resistance genes amongst isolates while virulence determinants have not been investigated. Therefore, the purpose of this study was to profile *S. aureus* isolates from sub-clinical mastitis affecting cattle/buffalo milk for their resistance as well as virulence properties using a combination of genotypic and phenotypic methods.

## Materials and methods

### Study population and sample collection

A total of 17 peri-urban dairy farms from two districts of Pakistan, falling in the Pothohar region, namely Islamabad and Rawalpindi were included in the study. These dairy farms were small-scale to medium-scale holdings having the number of animals ranging from 15 to to 45 per dairy farm. Within each farm, the number of collected samples varies from 5 to 15 depending on the size of the herd. A total of 278 milk samples were collected from these dairy farms in a sterile falcon tube (15 mL) during January 2021 to October 2021. All the animals were apparently healthy and animals suffering from clinical mastitis with visible symptoms were excluded from study. Each milk sample was collected by pooling streaks from all of the four quarters of the teat per animal. Thus, a single animal was designated the sampling unit in this study in accordance with ARRIVE guidelines. Teats were disinfected with 70% ethanol solution before sample collection to discourage contamination with teat microflora. The study was approved by the COMSATS ethical review board (letter no: CIIT/Bio/ERB/2020/12) and animals were handled following the specified regulations and reported in accordance with the ARRIVE and STROBE-vet guidelines. Milk samples were analyzed for mastitis using the California Mastitis Test (CMT) as described earlier^18^. A trained veterinarian, previously briefed about the study, was engaged to collect samples and conduct the CMT test. The samples were transported to the Animal Health Program of Animal Sciences Institute, National Agricultural Research Centre, Islamabad while maintaining cold conditions.. Samples were stored at 4 °C till further processing.

### Bacterial isolation and culture

Samples were enriched in Nutrient Broth (Oxoid, UK)^19–21^. A total of 9 ml of Nutrient broth was taken in a sterile universal bottle. 1 ml of milk sample was added in broth bottles and mixed. All milk samples were processed within 18 h after collection. The bottles were incubated overnight at 37 °C. Mannitol Salt Agar (MSA) (Oxoid, UK) was used to culture the samples. Isolates were next subjected to catalase test, coagulase test, and hemolysis for preliminary identification^22–24^. *S. aureus* was identified depending on the morphological properties observed on culture media and biochemical characteristics of isolate^25^.

### Molecular identification of isolates

Bacterial DNA was extracted by modified boiling method. In this method, colonies from isolated bacterial cultures were used. A few colonies were immersed and suspended in 200µL lysis buffer (10 mM EDTA (2 mL) + 50 mM Tris HCL (4 mL) + 3% Triton (4 mL)) taken in Eppendorf tube and boiled for 10 min at 96 ^o^C^26^. Immediately after boiling, the Eppendorf tubes are immersed in the ice bath for 5–10 min and centrifuged at 12,500 rpm for 5 min at room temperature. The DNA-containing supernatant is transferred to a new Eppendorf tube and processed for PCR or stored at − 20 °C for future use^27^. A 16S rRNA conserved gene was targeted in the confirmatory PCR for *S. aureus*. The thermal reaction mixture consisted of 2.5 µL of Taq buffer (Thermo Fisher Scientific, Waltham, USA), 1.5 µL of MgCl_2_ ((Thermo Fisher Scientific, Waltham, USA),, 1µL of each forward and reverse primer (Macrogen, Korea), 0.5 µL of dNTPs (Thermo Fisher Scientific, Waltham, USA), 0.3 µL of *Taq* DNA Polymerase (Thermo Fisher Scientific, Waltham, USA), and 15.2 µL of nuclease free water (Thermo Fisher Scientific, Waltham, USA),to make total volume of 22 µL. Total of 3 µL of template DNA was used in reaction.

### Detection of *mecA,* SCCmec typing and virulence profiling

Methicillin resistance-conferring gene *mecA* is present in most MRSA strains. The *mecA* gene encodes an additional penicillin-binding protein 2a (PBP 2a), which mediates cell wall synthesis in the presence of β-lactam antibiotics which is located on a mobile genetic element known as staphylococcal cassette chromosome mec (SCCmec)^28,29^. Hospital-acquired MRSA carries SCCmec type I, II and III whereas community acquired MRSA carries SCCmec type IV and V. The protocols used by Boyle et al. and McClure-Warnier were followed for the identification of SCCmec types I to V^30^. The Panton-Valentine leukocidin (PVL) gene is a well-recognized, toxin producing virulence factor carried predominantly by community-acquired MRSA strains and is cytotoxic for the bovine neutrophils^31^.

We employed a multiplex PCR for detection of both *mec*A and *Luk-PV* (PVL) genes. Isolates possessing the *mecA* gene were classified as MRSA. In addition to PVL, the *S. aureus* isolates were screened for the presence of several additional virulence factors which included hemolysins, including alpha (α) and beta (β), encoded by *hla* and *hlb* genes respectively; *nuc* encoding an extracellular thermostable nuclease^32^ and *fnb*A gene^33^. Furthermore, the isolates were screened for the presence of the bacterial cell wall component, capsular polysaccharide (CP) which helps in protection from phagocytic activity and enables immune system evasion. *Cap5* and *cap8* are most prevalent in *S. aureus* isolated from bovine and human infections^34^. Therefore their detection using multiplex PCR was performed as described earlier^35^. Similarly, the detection of *fnb*B, *clf*A, and *clf*B genes was performed using a multiplex PCR^33^. The Fishers exact test was used to compare the frequency of virulence genes among MRSA with that in MSSA isolates.

### Antibiotic sensitivity test (AST)

Kirby Bauer Disc diffusion method was used to determine the antimicrobial susceptibility profile of *S. aureus.* Isolates were cultured on Mueller–Hinton agar plates (Oxoid, UK) after calibrating bacterial suspension to 0.5 MacFarlands standard. Twelve antibiotics (Oxoid, UK) including Penicillin G (10 µg), Amoxicillin (10 µg), Cephradine (30 µg), Cefoxitin (30 µg), Cefotaxime (30 µg), Ceftazidime (30 µg), Cefepime (30 µg), Oxytetracycline (30 µg), Ciprofloxacin (5 µg), Gentamicin (10 µg), Imipenem (10 µg), and Azithromycin (15 µg) were applied. Plates were incubated for 24 h and the zone of inhibition was determined. Results were interpreted based on CLSI guidelines (Clinical Laboratory Standard Institute (CLSI) 2018). Antibiotic selection was based on clinical relevance in veterinary and human health^36^. Typically, cefoxitin, oxacillin disk diffusion, and mannitol salt agar screen, is used to characterize methicillin resistance but the gold standard is the genotypic detection of the *mecA* gene. Isolates showing inhibition zone < 22 mm for cefoxitin were considered for *mecA* screening and those positive for the gene were identified as methicillin-resistant *Staphylococcus aureus* (MRSA). Additionally, the AST data was comprehensively analyzed to characterize *S. aureus* isolates as multidrug-resistant (MDR: resistant to ≥ one agent in ≥ 3 antimicrobial classes), extensively drug-resistant (XDR: resistant to one or more antibiotics in all tested classes, except 1 or 2 classes), as previously described^37^.

## Results

### *S. aureus* prevalence in subclinical mastitis

A total of 278 milk samples were screened for subclinical mastitis by following the California Mastitis Test protocol as specified by the manufacturer. Of these, 173/278 (62%) animals were found positive for subclinical mastitis.

Out of 173 CMT positive samples 70 samples were culture positive for *Staphylococcus aureus.* Presumptive *S. aureus* colonies were sub-cultured and isolates were identified based on colony morphology and biochemical testing. The results showed a higher prevalence of CMT-positive samples and *S. aureus* isolates in Rawalpindi than in Islamabad. Distribution of CMT-positive cases and *S. aureus* isolation frequency also showed higher rates in cattle than buffalo. The district-wise and specie-wise CMT positive samples and *S. aureus* isolates are mentioned in Table 1.

**Table 1:** Distribution of CMT positive samples and *S. aureus* isolation frequency amongst various bovine species in two districts of Pothohar region.

Specie	Breed	Islamabad			Rawalpindi		
		No. of collected samples	CMT +	*S. aureus*	No. of collected samples	CMT +	*S. aureus*
Cattle	Friesian	25	19 (76%)	09 (36%)	17	11 (64.7%)	02 (11.7%)
	Holstien Friesian	15	10 (66.6%)	05 (33.3%)	27	19 (70%)	06 (22%)
	H. F Cross	13	07 (53.8%)	02 (15%)	11	10 (90.9%)	05 (45%)
	Dutch	04	03 (75%)	02 (50%)	12	10 (83%)	03 (25%)
	Mix	28	07 (25%)	02 (7%)	04	02 (50%)	01 (25%)
Buffalo	Neeli	25	12 (48%)	06 (24%)	12	09 (75%)	07 (58%)
	Neeli Ravi	08	04 (50%)	0	16	14 (87.5%)	05 (31%)
	Kundi	30	14 (46.6%)	05 (16.6%)	20	19 (95%)	09 (45%)
	Mix	03	03 (100%)	01 (33.3%)	08	0	0
Total	Cattle	85	46 (54%)	20 (23.5%)	71	52 (73%)	17 (23.9%)
	Buffalo	66	33 (50%)	12 (18%)	56	42 (75%)	21 (37.5%)
	Total	151	79 (52%)	32 (21%)	127	94 (74%)	38 (29.9%)

16SrRNA conserved gene sequence for *S. aureus* was targeted in the confirmatory PCR for confirmation of isolate identity. In total, 70/173 (40%) isolates were confirmed as *S. aureus*.

**Figure 1:**
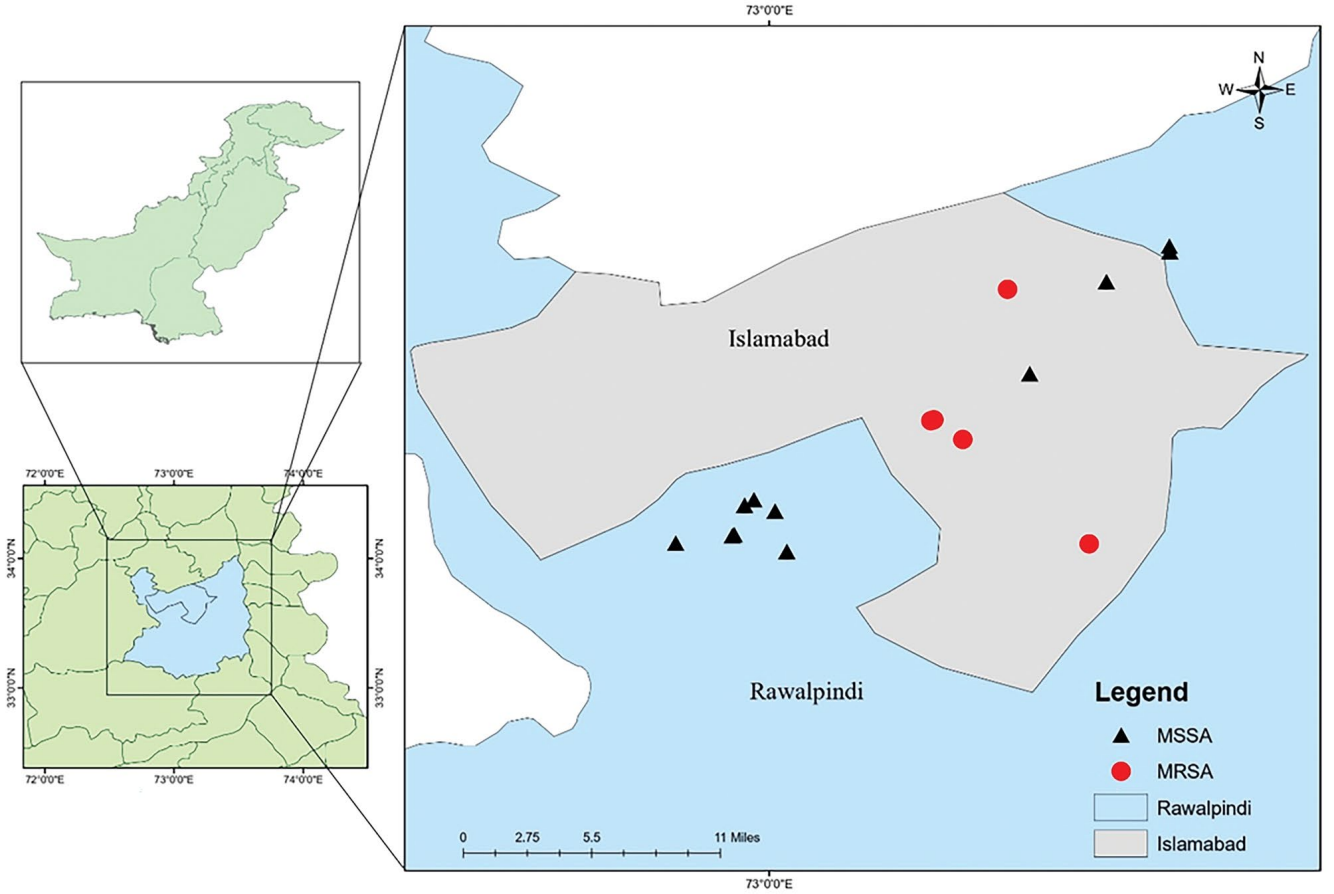
Map showing milk sampling locations in Pothohar Region with MRSA & MSSA distribution.

The results show a higher positivity rate in the Rawalpindi region due to less focus on cleaning practices while handling the animals, especially during milking. The geographical locations of MRSA-positive and MSSA-positive farms are shown in Fig. 1.

### Virulence gene profiling of *S. aureus*

PCR was carried out for the identification and detection of certain virulence genes *i.e., hla, hlb, fnbA, fnbB, clfA, clfB, cap5, cap8,* and *nuc* using primers established in the literature. Overall, the presence of selected genes was detected in all sub-clinical mastitis *S. aureus* isolates. In total, the highest number of isolates 58/70 (82%) were *hla* positive, followed by hemolysin *hlb* 47/70 (67%). Fibronectin binding protein *fnbA* was not detected in any isolate, while *fnbB* was detected in 5/70 (7%) isolates. *S. aureus* clumping factors *clfA, clfB* were present in 39/70 (55%) and 37/70 (52%) isolates respectively. Meanwhile, capsular polysaccharides *cap5* and *cap8* were detected in 32/70 (45%) and 6/70 (8.5%), isolates respectively. The thermonuclease *nuc* was present in 50/70 (71%) isolates (Table 2).

**Table 2:** Virulence genes distribution (% occurrence) amongst *S. aureus* isolates.

Virulence Genes	*S. aureus* (n = 70) %	MRSA (n = 15) %	MSSA (n = 55) %	*P* values^a^
*hla*	82	80	84	1.54
*hlb*	67	80	64	0.37
*nuc*	71	60	75	1.84
*clfa*	55	87	47	0.01
*clfb*	52	87	44	0.005
*fnbA*	0	0	0	–
*fnbB*	7	20	4	0.12
*LukPV*	5.7	20	2	0.05
*Cap5*	45	27	51	1.95
*Cap8*	8.5	7	9	1.55

^a^The frequency of virulence genes among MRSA was contrasted with that in MSSA isolates using Fishers exact test.

### MRSA identification and virulence characteristics

**Figure 2:**
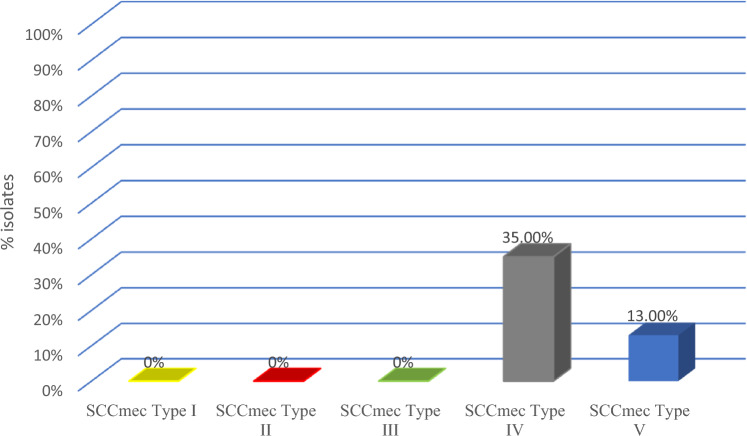
SCCmec Typing percentage among the LA-MRSA.

15 of the 70 (21%) isolates carried the *mecA* gene and were classified as MRSA. Among all Livestock Associated-MRSA strains, 33.3% of LA-MRSA carried SCCmec type IV whereas 13.3% of LA-MRSA carried SCCmec type V hence belonging to community acquired CA-MRSA which depicts that it was most likely transferred from farm handlers and workers who are in direct contact with livestock. Figure 2 depicts the overall analysis of SCCmec typing among LA-MRSA. Meanwhile, only 4/70 (5.7%) isolates possessed the *PVL* toxin gene. Virulence profiling showed the most common genes in MRSA isolates were *hla, hlb, clfA* and *clfB*. All the MRSA associated milk samples, especially the ones with *clfA* and *clfB* genes, were yellowish and had clots in it. Meanwhile, in MSSA isolates, the most common genes were *hla, hlb, nuc, clfA* and *cap5*. The frequency of *clfa, clfb* and *LukPV* genes among MRSA isolates was significantly higher than that for MSSA isolates (*P* < 0.05). All *S. aureus* isolates recovered in this study did not carry *fnbA* gene. Virulence genes distribution among MRSA and MSSA isolates is given in Table 3.

**Table 3:** Virulence genes distribution among MRSA & MSSA Isolates.

	*hla*	*hlb*	*nuc*	*clfa*	*clfb*	*fnbA*	*fnbB*	LukPV	*Cap5*	*Cap8*
MRSA	12/15 (80%)	12/15 (80%)	9/15 (60%)	13/15 (87%)	13/15 (87%)	0/15	3/15 (20%)	3/15 (20%)	4/15 (27%)	1/15 (7%)
MSSA	46/55 (84%)	35/55 (64%)	41/55 (75%)	26/55 (47%)	24/55 (44%)	0/55	2/55 (4%)	1/55 (2%)	28/55 (51%)	5/55 (9%)
Total	58/70	47/70	50/70	39/70	37/55	0/70	5/70	4/70	32/70	6/70

### Antibiotic Susceptibility Test (AST)

**Figure 3:**
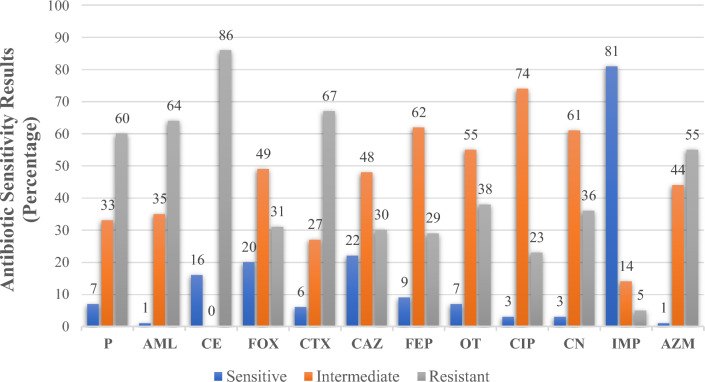
Antibiotic resistance profiling of *S. aureus* isolates using Kirby-Bauer disc diffusion test.

The highest number of *S. aureus* isolates were resistant against penicillin class (penicillin (60%) and amoxicillin (64%)), 1st generation cephalosporins (cephradine (86%)), 3rd generation cephalosporins (cefotaxime (67%)), and macrolides (azithromycin (55%)). On the other hand, relatively low resistance was exhibited against 2^nd^ generation cephalosporins (cefoxitin (31%)), 3rd generation cephalosporins (ceftazidime (30%)), 4th generation cephalosporins (cefepime (29%)), tetracycline (oxytetracycline (38%)), aminoglycosides (gentamicin (36%)), and fluoroquinolones (ciprofloxacin (23%)). Few isolates exhibited resistance against carbapenem (imipenem (5%)). Details of antibiotic resistance profiling are shown in Fig. 3. 37/70 (52%) of the isolates were resistant to 3 or more antibiotics and categorized as multidrug-resistant (MDR). Approximately, 53% of MRSA isolates and 65% of MSSA isolates were MDR (Table 4).

**Table 4:** Common antibiotic susceptibility and resistance patterns observed in *S. aureus* isolates.

	Common Susceptible Antibiotics	Common Resistant Antibiotics	MDR
All isolates	Ciprofloxacin (CIP) Gentamicin (CN) Cefepime (FEP) Ceftazidime (CAZ) Imipenem (IPM) Oxytetracycline (OT) Cefoxitin (FOX)	Penicillin G (P) Amoxicillin (AML) Cephradine (CE) Cefotaxime (CTX) Azithromycin (AZM)	37/70 (52%)
MRSA (15/70)	Cefoxitin (FOX) Ceftazidime (CAZ) Cefepime (FEP) Oxytetracycline (OT) Gentamicin (CN) Imipenem (IPM) Ciprofloxacin (CIP)	Amoxicillin (AML) Penicillin G (P) Cephradine (CE) Cefotaxime (CTX)	8/15 (53%)
MSSA (55/70)	Cefepime (FEP) Gentamycin (CN) Ciprofloxacin (CIP) Cefoxitin (FOX) Ceftazidime (CAZ) Oxytetracycline (OT) Imipenem (IPM)	Penicillin G (P) Amoxicillin (AML) Cephradine (CE) Cefotaxime (CTX) Azithromycin (AZM)	36/55 (65%)

Antibiotics most isolates were susceptible to included ciprofloxacin, gentamicin, oxytetracycline, cefepime, ceftazidime, and imipenem, while common resistant antibiotics were penicillin, amoxicillin, cephradine, cefotaxime, and azithromycin. The antibiotic susceptibility pattern was similar amongst MSSA and MRSA isolates. Meanwhile, MRSA isolates were resistant to penicillin G, amoxicillin, cephradine, and cefotaxime, which aligns with their methicillin-resistant nature. On the other hand, MSSA showed additional resistance against azithromycin (Table 4).

## Discussion

Mastitis represents a significant challenge for the dairy industry in Pakistan, resulting in substantial production losses. Mastitis has been recognized as one of the most economically important diseases affecting dairy animals worldwide^38^. Meanwhile, subclinical mastitis also leads to decreased milk yield and poor milk quality and often goes undetected in herds leading to widespread dissemination. To determine the prevalence of subclinical mastitis in dairy herds of Islamabad and Rawalpindi 278 pooled milk samples were collected from small to medium scale holdings in both districts. 74% of milk samples were found positive for subclinical mastitis using the CMT from Rawalpindi as compared to 52% from Islamabad. Less incidence of sub clinical mastitis in Islamabad may be attributed to better hygienic conditions and literary status of farmers while in Rawalpindi high incidence of mastitis may be correlated with poor hygienic conditions at farm, as the chances of sub clinical mastitis increases due to poor animal hygiene^39,40^. In our study, overall subclinical mastitis prevalence was found to be 62%. This is higher than the prevalence rates reported in earlier studies^5,41^, and corresponds with high prevalence of subclinical mastitis reported earlier from the Pothohar region which includes Islamabad, Rawalpindi, Attock, Jehlum, and Chakwal districts^42^. These results depict the growing burden of subclinical mastitis within dairy animals in this region. Similar to previous reports we also found high prevalence (40%) of *S. aureus* in subclinical mastitis milk samples underlining its importance as the causative agent of bovine mastitis^5,7,41,43^.

Methicillin resistant *Staphylococcus* was first confirmed as the causative agent of mastitis in dairy cattle in 1972^44^. The presence of MRSA in bovine mastitis poses a significant risk to farmworkers, including veterinarians, and can lead to severe intra-herd infections^45,46^. Although previous studies have reported a low prevalence of MRSA, ranging up to 4.3%, in Asia, a relatively higher prevalence of up to 6.47% has also been documented^47^. In our study we observed a much higher MRSA prevalence (21%) amongst *S. aureus* isolates from cases of bovine subclinical mastitis. Similarly, a high prevalence of MRSA (19.6%) was reported in another province of Pakistan, Khyber Pakhtunkhwa, highlighting the increasing prevalence of MRSA as a causative agent of bovine mastitis^7^. This trend could be attributed to the neglect of cleanliness practices during milking by milkers and farm management.. MRSA prevalence is linked to farm population, farming techniques, disinfectants used, and animal commerce^48^. Our results also showed that the majority of the MRSA isolates carried SCCmec type IV, while 13% of the isolates carried *mec* type V, which is associated with community acquired MRSA. The presence of CA-MRSA particularly *mec* (SCCmec) types IV and IVa has also been reported from Korea amongst mastitis affected dairy cattle^49^. The carriage of mec type V is often associated with CA-MRSA, suggesting the potential for transmission through farmer handling practices.

*S. aureus* produces several virulence factors that help in its pathogenesis, resulting in mastitis^16^. There are two major classes of these virulence factors: surface associated structural components and secreted virulence factors, which help *S. aureus* evade host immune responses and cause chronic infection^50^.

The Panton-Valentine leukocidin (PVL) gene is a common, well-recognized, toxin-producing virulence factor of *S. aureus*. PVL is most cytotoxic for bovine neutrophils^31^. This toxin is also commonly associated with the methicillin-resistant *S. aureus*^51^. The presence of the PVL gene in methicillin-resistant *S. aureus* from other animals has been previously reported. In this study. All the PVL positive isolates were also positive for the *mecA*. In total, 5.7% of isolates tested positive for *Luk-PV* indicating the presence of PVL toxin with a higher occurrence in MRSA than MSSA, which is in accordance with previous reports of PVL gene occurrence amongst *S. aureus* and MRSA bovine isolates respectively^52,53^.

Haveri et al. (2008) presented that *fnbA* and *fnbB* genes coexist in the predominant PFGE types of *S. aureus* strains isolated from bovine intramammary infections^54^. Meanwhile, *fnbB* is more common than *fnbA* in *S. aureus* isolates from subclinical mastitis^55^. Our study also depicted a higher prevalence of *fnbB,* while *fnbA* was not detected among our isolates. The ability of adhesion to fibronectin is important during several steps of the disease process because fibronectin is a ubiquitous host protein present in soluble form in the blood and in fibrillar form in cellular matrices^56^.

The ability of *S. aureus* to bind to fibrinogen and fibrin is believed to be an important factor in the initiation of foreign-body and wound infections^57^. A receptor for fibrinogen called the clumping factor (*ClfA*) is located on the surface of *S. aureus* cells. The affinity is very high, and clumping occurs in low concentrations of fibrinogen^58,59^. Previously, the cell wall-anchored protein clumping factor B (ClfB) has been demonstrated to play a crucial role in facilitating nasal colonization of *S. aureus*. This occurs through high-affinity interactions with the cornified envelope within the anterior nares of humans.. *ClfB* exerts its effect in the early stage of infection, and its interaction with loricrin appears to play a role during pathogenesis and making this a vital virulence factor for vaccine formulation^60^. Our data shows the prevalence of fibrinogen-associated clumping factors *clfA* and *clfB* to be 55% and 52%, respectively, which is similar to previous reports^60^.

The capsular polysaccharide or capsule is a cell wall bacterial component that provides protection to bacterial cell against the phagocytic activity of immune cells. In previous studies the *cap5* gene frequency was much higher (42–70%) than *cap8* (6.6%) in bovine subclinical mastitis isolates^61,62^. In this study *cap5* was detected among 45% and *cap8* was detected in 8.5% of bovine subclinical mastitis isolates.

Extra cellular thermostable nuclease (*nuc* gene) is a common enterotoxin and virulence factor produced by *S. aureus,* which helps in evasion of immune cells of host^63^. In a recent study 31% of isolates had the *nuc* gene^64^. In this study *nuc* gene was detected and confirmed in 71% of *S. aureus* isolates.

*Hla* and *hlb* encode for hemolysin toxins and were detected in 82% and 67% of bovine subclinical mastitis isolates respectively. Similar prevalence has been reported from China and Egypt^15,65,66^. *Hla* and *Hlb* have been also reported as the most common toxin genes among *S. aureus* isolates in Pakistan^67^. Hemolysins, attack cell membranes, cause platelet damage, destruction of lysosome, necrosis, and ischemia. *Hla* (α hemolysin) is a beta-barrel pore-forming toxin that disrupts the cell membrane causing irreversible osmotic changes, resulting in cell-death by apoptosis^68^. Meanwhile Beta hemolysin (*Hlb*) is encoded by a lysogenic bacteriophage; in itself, it cannot destroy most cell types, but it exposes vulnerable cells to other lytic proteins, such as *Hla* and leukocidins like PVL^69^.

Antimicrobial resistance is fast becoming a major health threat. Multidrug resistant *S. aureus* infections are becoming common in recent times. It must be noted that failure to treat chronic *S. aureus* infection can lead to the bacterium penetrating the mammary gland tissue forming an abscess and eventual scar tissue formation^70^. In the current study we observed highest number of resistant isolates against the class penicillin (penicillin g and amoxicillin) as reported earlier from Pakistan^5,12^ as well as globally^71–73^. Subclinical bovine mastitis *S. aureus* isolates in this study also showed very high resistance against 1st generation cephalosporins (cephradine) and moderate resistance against the 3rd generation cephalosporins (cefotaxime), as has been observed earlier^11^.

Macrolide (azithromycin) resistance was also observed among 55% of *S. aureus* isolates asreported previously from the region^12^.

On the other hand, all the *S. aureus* isolates were sensitive towards other antibiotic classes including 2^nd^ and 4th generation cephalosporins (cefoxitin & cefepime), tetracyclines (oxytetracycline), fluoroquinolones (ciprofloxacin), aminoglycosides (Gentamicin), and carbapenem (imipenem). These results are consistent with previous observations^11^ and present alternatives for therapy. Notably, gentamicin and enrofloxacin antibiotics are listed in the approved list of antimicrobial agents approved for veterinary medicine by the World Health Organization and World Organization for Animal Health^74^.

The use of antibiotics for the treatment of mastitis helps to a large extent in avoiding economic losses from the disease. Most of the *S. aureus* strains are normally sensitive to majority of antibiotics but antibiotic resistance is becoming a problem, so it is advisable to perform antibiotic sensitivity test to minimize the hazard of drug resistance and to avoid economic loss on treatment. In this study almost 52% of the isolates were found to be multidrug resistant. These figures are alarming and reflect the unsupervised usage of antibiotics in veterinary practices.

## Conclusions

Overall prevalence of sub-clinical mastitis was high in Pothohar region of the country with a high frequency of *S. aureus* isolation from the affected teat. Virulence profiling of *S. aureus* isolates showed high prevalence of hemolysins *hla*, *hlb*, and factors associated with host immune evasion. Meanwhile the leucocidin, PVL was present in few isolates that also carried the methicillin resistance gene *mecA*. We report high prevalence of MRSA compared to previous reports indicating a possible selection pressure for *mecA* and PVL positive isolates. Antibiotics sensitivity test shows that all the strains of *S. aureus* were sensitive to ciprofloxacin, gentamycin, and oxytetracycline. Managers of dairy herds should adopt good milking technique, to limit bacterial spread amongst dairy animals.

### Supplementary Information

Supplementary Information.

## Data Availability

The data that support the findings of this study are not openly available due to reasons of sensitivity and are available from the corresponding author upon reasonable request.
