# Supplementary material for: Virulence and resistance profiling of Staphylococcus aureus isolated from subclinical bovine mastitis in the Pakistani Pothohar region
Source: Sci Rep. 2024 Jun 24;14:14569. doi: 10.1038/s41598-024-65448-9 (PMC11196630; doi:10.1038/s41598-024-65448-9)
Supplement: Supplementary file 1 — Supplementary Information. [file 41598_2024_65448_MOESM1_ESM.docx]

**Supplementary**

**Table 1.** Primer Sequence, Length and PCR conditions for the detected genes.

| **Sr. No** | **Gene** | **Primer Sequence** | **Length (bp)** | **PCR Conditions** | **Reference** |
| --- | --- | --- | --- | --- | --- |
| 1 | **16SrRNA** | ATTAGATACCCTGGTAGTCCACGCC-F  CGTCATCCCCACCTTCCTCC-R | 409 | PCR was performed following these conditions: Initial denaturation 94^o^C for 5 min; Denaturation 94^o^C for 45 seconds; Annealing 60^o^C for 45 seconds; Amplification 72^o^C for 30 seconds; Final extension 72^o^C for 10 minutes then resting at 22^o^C and run the thermocycler for 30 cycles. | (Fang et al., 2018) |
| 2 | ***Luk*PV** | ATCATTAGGTAAAATGTCTGGACATGATCCA-F  GCATCAASTGTATTGGATAGCAAAAGC-R | 433 | Initial denaturation 95^o^C for 5 min; Denaturation 95^o^C for 1 min; Annealing 55^o^C for 1 min; Amplification 72^o^C for 1 min 30 seconds; Final extension 72^o^C for 10 minutes for 30 cycles. | (Makgotlho et al., 2009) |
| 3 | ***mec*A** | GTAGAAATGACTGAACGTCCGATAA-F  CCAATTCCACATTGTTTCGGTCTAA-R | 310 |  |  |
| 4 | ***Hla*** | TGCCGCAGATTCTGATATTAA-F  TTTCTGAAGAACGATCTGTCCA-R | 845 | Initial denaturation at 94^o^ C for 5 min; 35 cycles of 94^o^ C for 30s, 51.5^o^ C for 30s, and 72^o^ C for 30–90s depending on the PCR product length and a final extension at 72^o^C for 7 min. | (Ote et al., 2011) |
| 5 | ***Hlb*** | GCGGTTGTGGATTCGATAAT-F  GGCTTTGATTGGGTAATGATC-R | 524 | Initial denaturation at 94^o^ C for 5 min; 35 cycles of 94^o^ C for 30s, 50.9^o^ C for 30s, and 72^o^ C for 30–90s depending on the PCR product length and a final extension at 72^o^C for 7 min. | (Ote et al., 2011) |
| 6 | ***fnbA*** | GTGAAGTTTTAGAAGGTGGAAAGATTAG-F  GCTCTTGTAAGACCATTTTTCTTCAC-R | 643 | Initial denaturation at 94^o^ C for 5 min; 25 cycles of 94^o^ C for 1min, 55^o^ C for 1min, and 72^o^ C for 1min and a final extension at 72^o^C for 10 min. | (Tristan et al., 2003) |
| 7 | ***fnbB*** | GTAACAGCTAATGGTCGAATTGATACT-F  CAAGTTCGATAGGAGTACTATGTTC-R | 524 |  |  |
| 8 | ***clfA*** | ATTGGCGTGGCTTCAGTGCT-F  CGTTTCTTCCGTAGTTGCATTTG-R | 292 |  |  |
| 9 | ***clfB*** | ACATCAGTAATAGTAGGGGGCAAC-F  TTCGCACTGTTTGTGTTTGCAC-R | 205 |  |  |
| 10 | ***Cap5*** | GTCAAAGATTATGTGATGCTACTGAG-F  ACTTCGAATATAAACTTGAATCAATGTTATACAG-R | 361 | Initial denaturation at 94^o^ C for 5 min; 25 cycles of 94^o^ C for 30s, 55^o^ C for 30s, and 72^o^ C for 1min and a final extension at 72^o^C for 5 min. | (Verdier et al., 2007) |
| 11 | ***Cap8*** | GCCTTATGTTAGGTGATAAACC-F  GGAAAAACACTATCATAGCAGG-R | 173 |  |  |
| 12 | ***nuc*** | GCGATTGATGGTGATACGGTT-F  AGCCAAGCCTTGACGAACTAAAGC-R | 270 | A total of 37 PCR cycles were run under the following conditions: DNA denaturation at 94°C for 1 min, primer annealing at 55°C for 30s, and DNA extension at 72°C for 90s. After the final cycle, the reaction was terminated by keeping it at 72°C  for 3.5 min. | (Brakstad et al., 1992) |
| 13 | ***α3*** | R-TAAAGGCATCAATGCACAAACACT | 937 | Total of 34 PCR cycles were run under following conditions:  Initial Denaturation at 95˚C for 4 min  Denaturation at 95˚C for 1min, primer annealing at 53˚C for 1 min, extension at 72˚C for 1 min 30 sec and final extension at 72˚C for 4 min | (Boye et al. 2007) |
| 14 | ***β*** | F-ATTGCCTTGATAATAGCCYTCT |  |  |  |
| 15 | ***5RmecA*** | F-TATACCAAACCCGACAACTAC | 359 |  |  |
| 16 | ***5R431*** | R-CGGCTACAGTGATAACATCC |  |  |  |
| 17 | ***ccrC*** | F-CGTCTATTACAAGATGTTAAGGATAAT  R-CCTTTATAGACTGGATTATTCAAAATAT | 518 |  |  |
| 18 | ***1272*** | F- GCCACTCATAACATATGGAA | 415 |  |  |

**Supplementary Figures**


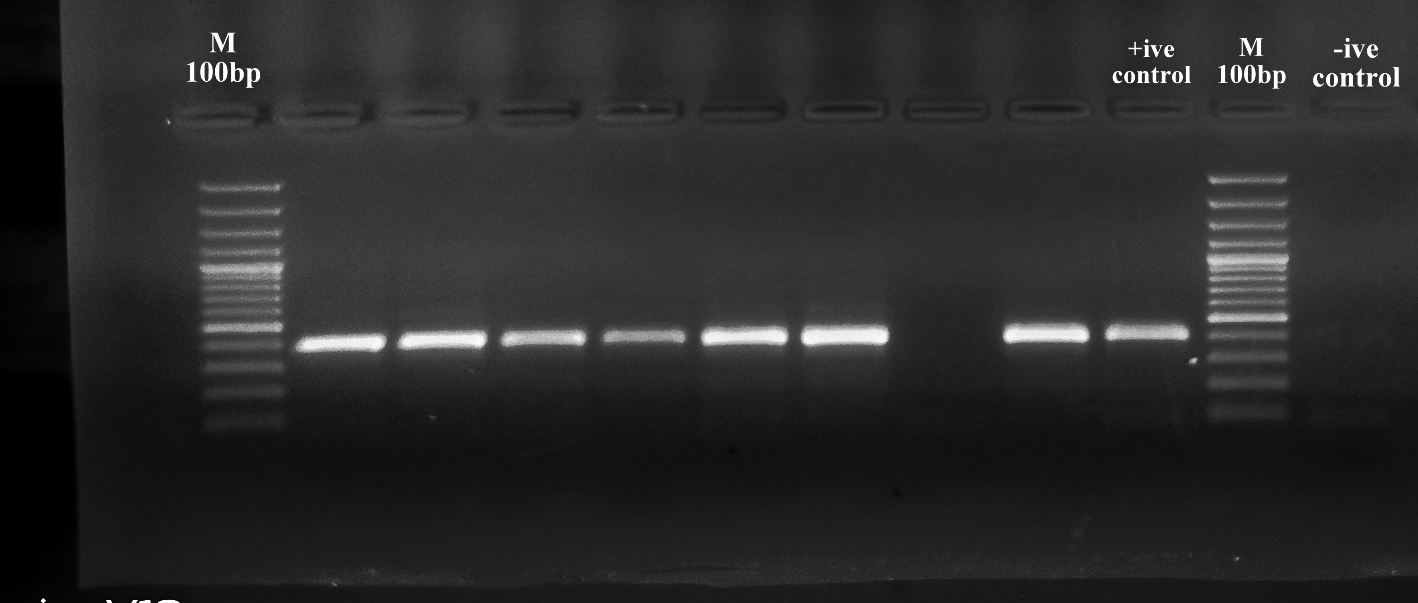


409bp

Supplementary Figure 1. PCR products of 409bp for 16SrRNA gene of *S. aureus*


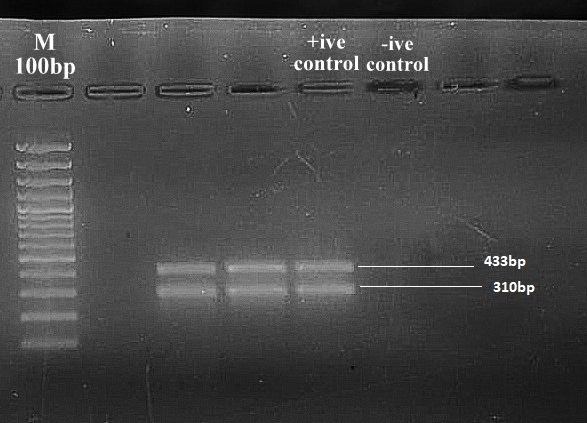


Supplementary Figure 2: PCR Products for *mecA* (310bp) and LukPVL (433bp) genes with 100bp gene marker.


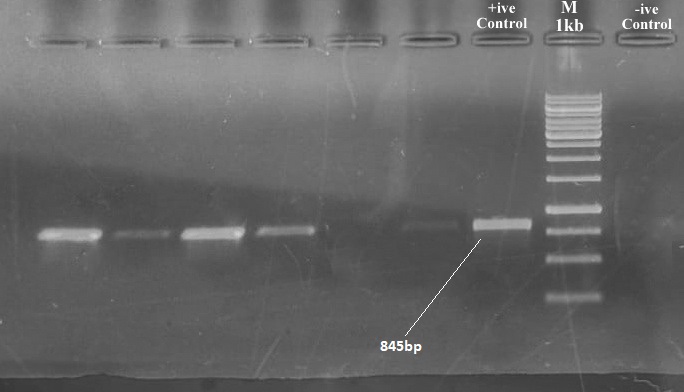


Supplementary Figure 3: PCR products for *hla* gene (845bp) with 1kb gene marker.


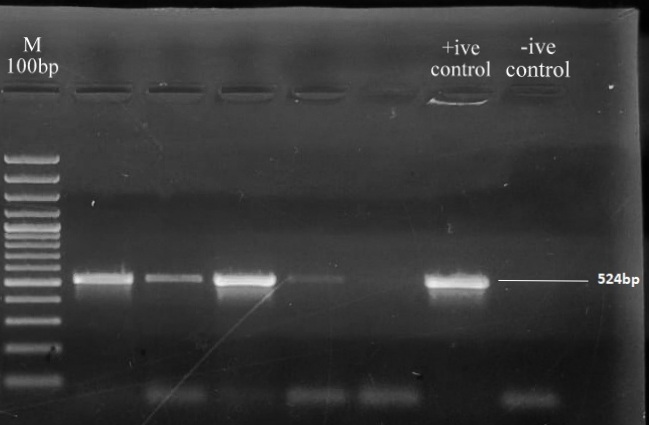


Supplementary Figure 4: PCR Product of *hlb* gene (524bp) with 100bp gene marker.


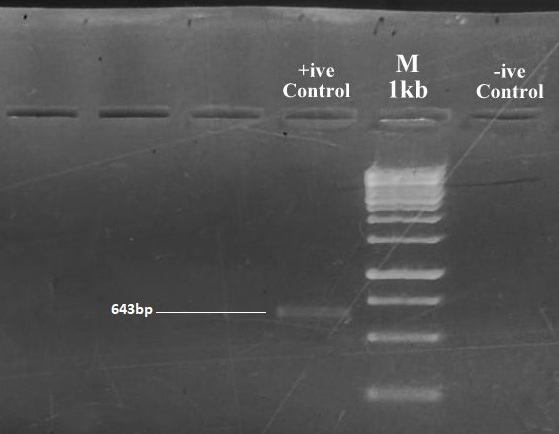


Supplementary Figure 5: PCR Product of *fnbA* gene (643bp) with 1kb gene marker.


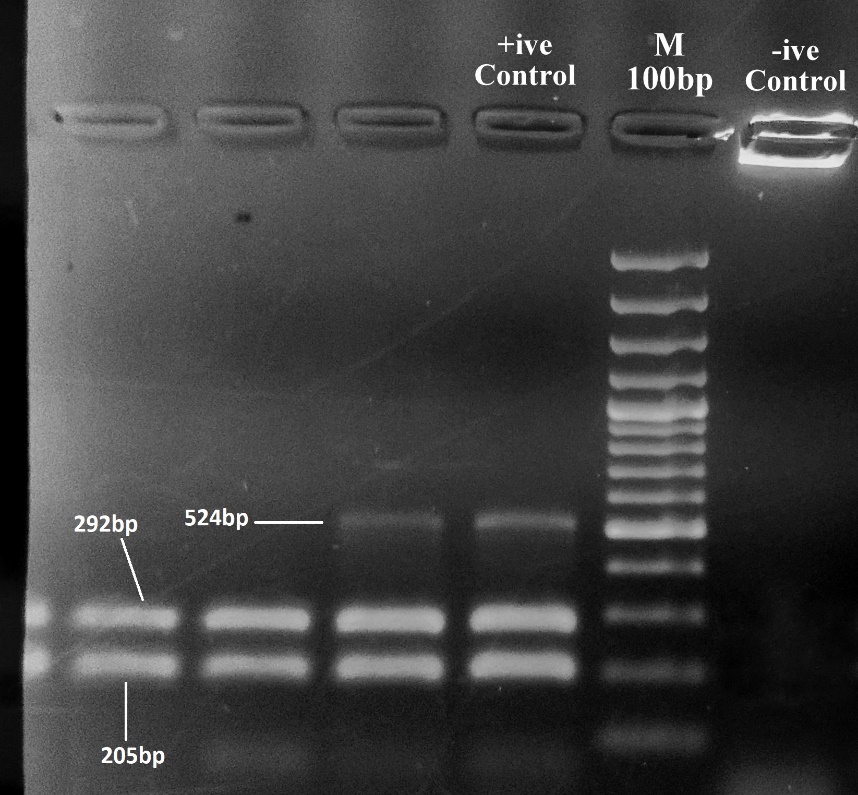


Supplementary Figure 6: PCR Product for *clfB* gene (205bp), *clfA* gene (292bp), & *fnbB* gene (524bp) along with 100bp gene marker.


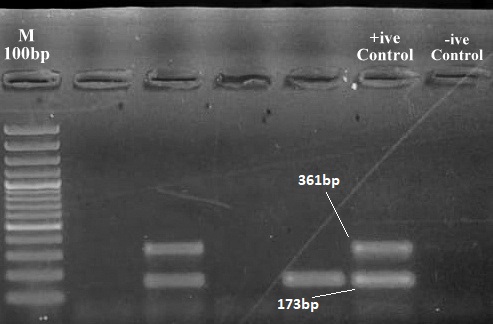


Supplementary Figure 7: PCR Products for *Cap8* gene (173bp) & *Cap5* gene (361bp) along with 100bp gene marker.


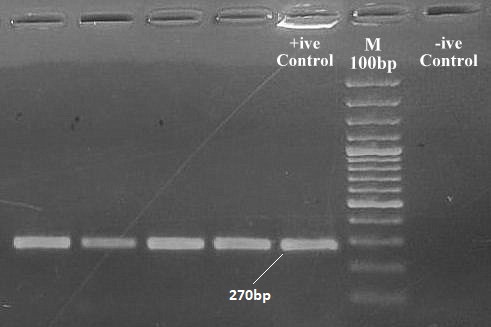


Supplementary Figure 8: PCR Product for *nuc* gene (270bp) along with 100bp gene marker.


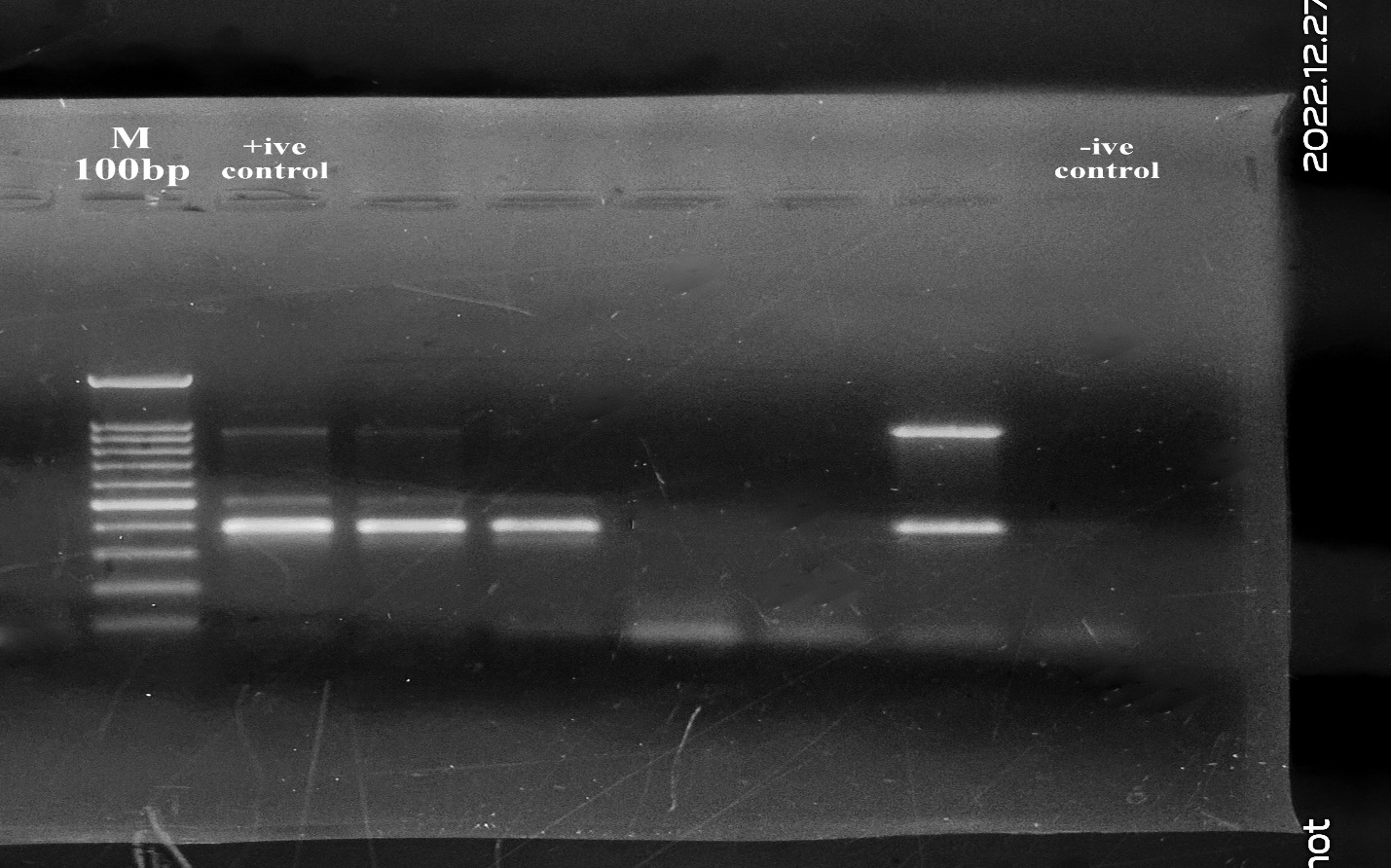


Supplementary Figure 9: SCCmec typing of MRSA obtained from livestock sources
